# Cytomegalovirus Retinitis Associated with Lenalidomide Use for Multiple Myeloma in an Immunocompetent Patient

**DOI:** 10.1155/2019/3516394

**Published:** 2019-02-25

**Authors:** Matthew K. Adams, Christina Y. Weng

**Affiliations:** Department of Ophthalmology, Baylor College of Medicine, 6565 Fannin Street, NC-205, Houston, TX 77030, USA

## Abstract

**Purpose:**

The aim of this report is to present a case of cytomegalovirus (CMV) retinitis in an immunocompetent patient using lenalidomide.

**Methods:**

Case report with fundus photography, spectral-domain optical coherence tomography, and fluorescein angiography imaging.

**Results:**

A 55-year-old male with history of multiple myeloma treated with lenalidomide presented with blurriness and floaters in his right eye and was found to have vitreous biopsy-confirmed CMV retinitis. The patient was treated with pars plana vitrectomy, oral valganciclovir, and intravitreal foscarnet. More than one year later, the patient was doing well with visual acuity of 20/25 and no recurrence of retinitis.

**Conclusion:**

This represents the second report of CMV retinitis associated with lenalidomide therapy. It suggests that even immunocompetent patients can be affected by CMV retinitis in the context of lenalidomide treatment. It is critical that patients being treated with lenalidomide receive prompt evaluation if they develop ophthalmic symptoms.

## 1. Introduction

Cytomegalovirus (CMV) infection typically occurs in immunocompromised patients and can present as a primary infection, reinfection, or reactivation. While generally asymptomatic in immunocompetent individuals, CMV can lead to serious pathology in those who are immunocompromised, such as transplant recipients, patients with immunodeficiency disorders, and those on immunosuppressive treatment [1].

Lenalidomide (Revlimid®; Celgene, Summit, NJ), an analogue of thalidomide, is an FDA-approved treatment for multiple myeloma, myelodysplastic syndrome, and mantle cell lymphoma. Its mechanism of action is complex and includes immunomodulation, antiangiogenic effects, and direct cytotoxic activity [2, 3]. While it is thought to have a more favorable side-effect profile than its parent drug, thalidomide, it still has potential serious adverse effects that include birth defects, neutropenia, and thrombocytopenia [4].

CMV retinitis is most commonly seen in AIDS patients with a CD4+ count < 50 cells/*µ*L; it is less commonly seen in patients with other causes of immunosuppression [5]. However, it has been reported once before in an immunocompetent multiple myeloma patient following lenalidomide therapy [6]. Here, we present the second reported case of CMV retinitis in a similar patient who was treated with lenalidomide; differences in management between the first reported case and ours are discussed.

## 2. Case Report

A 55-year-old male with multiple myeloma on his eighth cycle of chemotherapy with bortezomib, lenalidomide (recently decreased to 10 mg po daily from 25 mg po daily), and dexamethasone presented with a two-week history of worsening blurriness and floaters in his right eye. An outside provider noted panuveitis and retinal whitening on examination. Anterior chamber paracentesis was negative for CMV, HSV-1, HSV-2, VZV, and Toxoplasmosis. Oral valacyclovir and topical steroids and cycloplegics were started for presumed acute retinal necrosis, and the patient was referred to our institution for further diagnostic work-up and management. Of note, recent serum laboratory values revealed a normal white blood cell count (9.41 k/*µ*L; range 4.5–11 k/*µ*L) and neutrophilic profile (71.0%; range 39–69%) with negative CMV antigen and aerobic blood culture; lambda light chain immunoglobulins were significantly elevated (67.29 mg/L, range 5.7–26.3 mg/L) and alpha-2 globulin proteins were abnormally high on serum electrophoresis (0.93 g/dL, range 0.58–0.84 g/dL) as expected given his disease.

**Figure 1:**
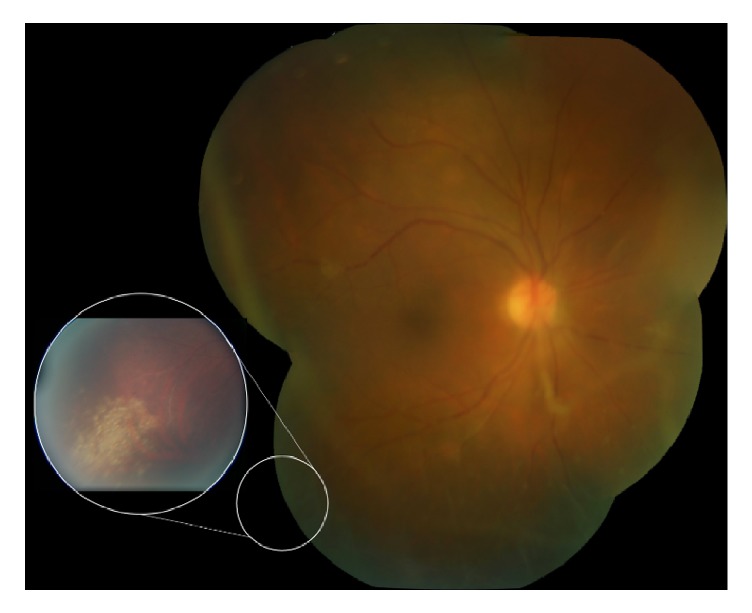
Color fundus photograph of the right eye shows vitreous haze, vascular sheathing, and granular retinal whitening in the inferotemporal periphery (inset).

**Figure 2:**
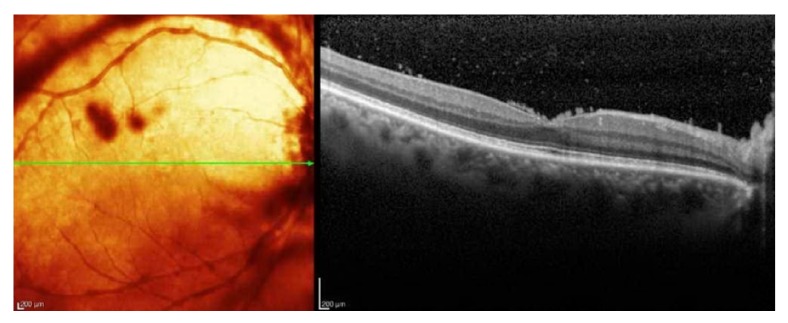
Spectral-domain OCT of the right eye demonstrates vitreous debris overlying a well-preserved foveal contour.

**Figure 3:**
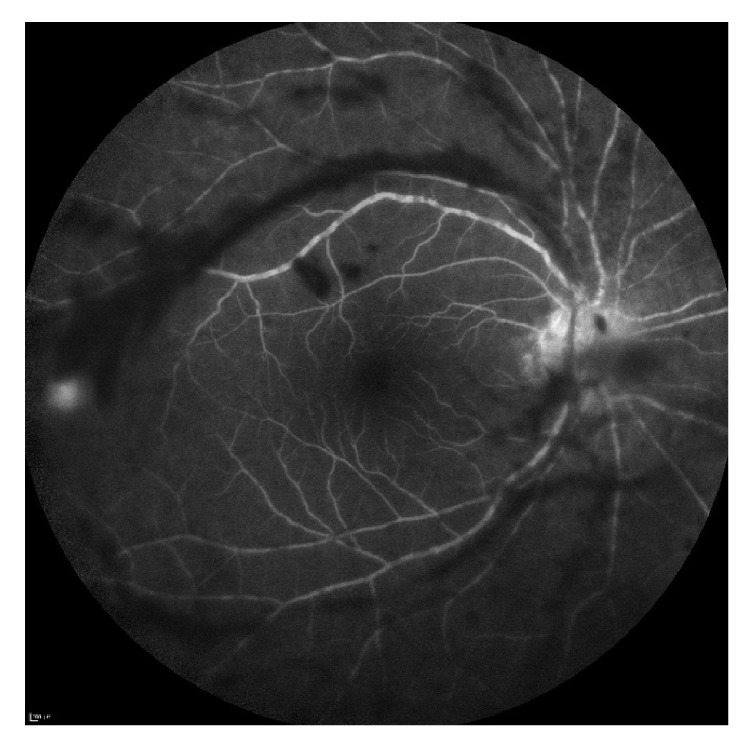
Fluorescein angiography of the right eye reveals irregular filling defects and blockage from overlying vitreous debris; of note, the inferotemporal area of retinal whitening is not captured in this image.

Best-corrected visual acuity (BCVA) was 20/60 in the right eye, 20/20 in the left. Pupils and intraocular pressures were normal. Slit lamp examination of the right eye revealed mild anterior chamber flare without cell, mild cataract, and 3+ cell in the anterior vitreous. Dilated funduscopic examination revealed vitreous haze, diffuse vascular sheathing, and a poorly-defined area of inferotemporal retinal whitening without associated hemorrhage (Figure 1). The left eye was normal. Spectral-domain optical coherence tomography of the right eye showed no significant abnormalities aside from overlying hyperreflective vitreous debris (Figure 2). Fluorescein angiography demonstrated irregular vascular filling and blockage from vitreous debris (Figure 3).

**Figure 4:**
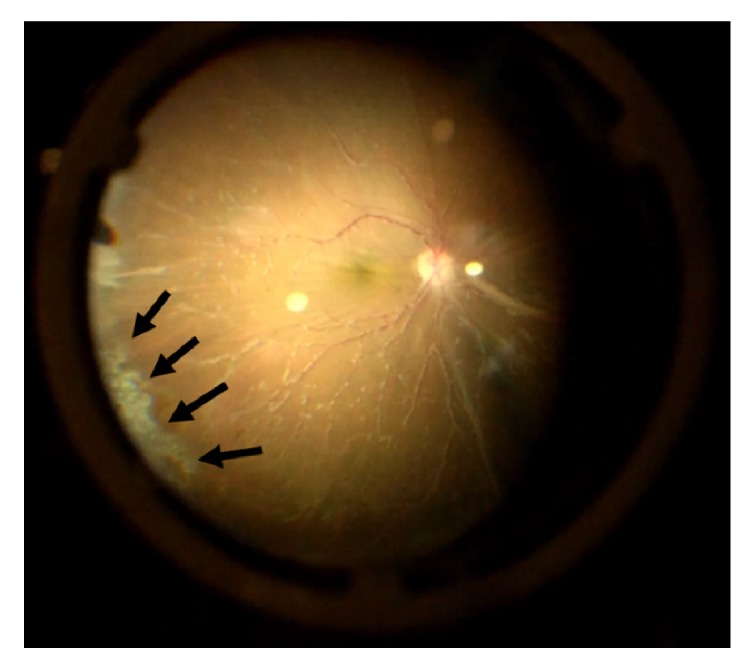
Intraoperative image of fundus reveals diffuse vascular sheathing with perivascular inflammatory aggregates and an area of granular retinal necrosis and whitening (arrows) involving the inferotemporal periphery.

Given the previous negative anterior chamber tap, worsening clinical exam, and high suspicion for infectious retinitis, the decision was made to proceed with a diagnostic vitrectomy. Diluted and undiluted vitreous biopsy were obtained via a 23-gauge pars plana vitrectomy. Intraoperatively, extensive retinal whitening and granular necrosis were observed along with diffuse vascular sheathing and perivascular inflammatory aggregates (Figure 4). Endolaser was placed 360 degrees prophylactically. The vitreous biopsy was sent for gram stain, cytology (to rule out malignant infiltration), fungal culture, Toxoplasmosis, AFB, VZV, EBV, CMV, HSV-1, HSV-2, and RPR testing.

**Figure 5:**
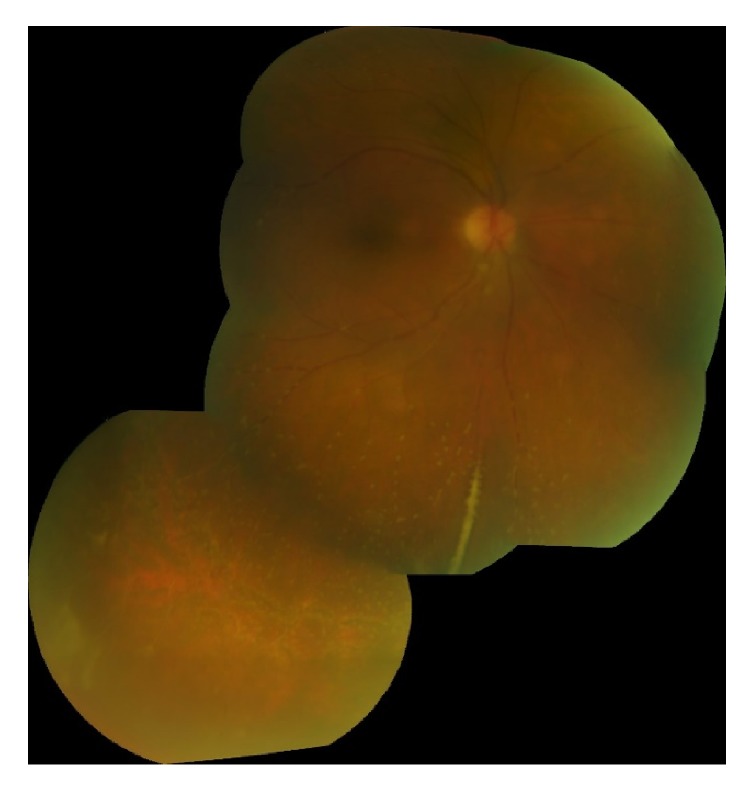
Fundus photograph from postoperative week 1 demonstrates improving vasculitis and retinitis.

**Figure 6:**
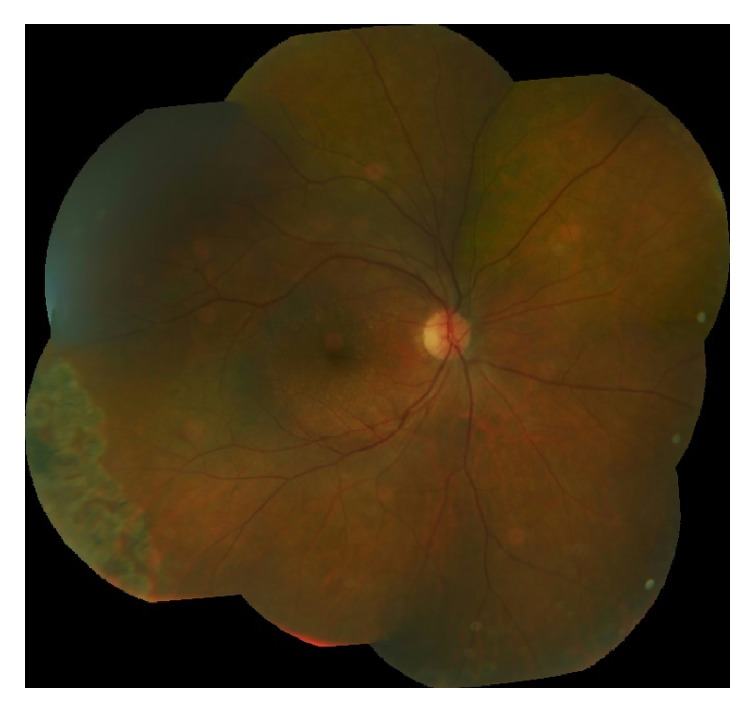
Fundus photograph more than one year following initial presentation shows resolution of retinitis with laser scars in the periphery.

On postoperative day 2, the vitreous PCR returned positive for CMV; all other tests were negative. The patient was switched from oral valacyclovir to valganciclovir 900 mg po twice daily. By postoperative day 3, the patient's visual acuity declined to 20/200 and there was an increase in anterior chamber cell. Hence, intravitreal foscarnet (2.4 mg/0.1 cc) was injected. At the postoperative week 1 visit, visual acuity had not improved, but the area of retinitis appeared less active (Figure 5). Repeat intravitreal foscarnet was given. Improvement continued over the first month with complete resolution of the patient's uveitis, vasculitis, and retinitis. By postoperative month 2, BCVA had improved to 20/63 without evidence of active disease. Five months after the vitreous biopsy, the patient remained without signs of intraocular infection with BCVA 20/50, mostly limited by cataract. Following cataract extraction one year later, his visual acuity was 20/25 with stable fundus findings (Figure 6).

## 3. Discussion

Cytomegalovirus retinitis is thought to result from reactivation of a latent infection and is typically only seen in those with a compromised immune system. Multiple myeloma itself, although capable of causing an immunocompromised state by affecting cellular immunity, has never been reported to cause CMV retinitis [7]. Given that our patient was essentially immunocompetent (by definition of a normal white blood cell count and differential) along with the fact that lenalidomide-associated CMV retinitis has been previously reported, it is reasonable to assume that lenalidomide played a role in this patient's development of CMV retinitis. Notably, none of his other medications have been associated with this condition.

The first case of CMV retinitis following lenalidomide treatment was presented by Lim et al. in 2013 [6]. Similar to this case, CMV retinitis presented in their patient with multiple myeloma, on lenalidomide therapy, and in the absence of detectable systemic CMV titers. Lenalidomide can cause myelosuppression, but, like the previously-reported patient, our patient had a normal white blood cell count without neutropenia [4]. Our patient also received multiple blood draws during his treatment period and never had detectable CMV antigen. This lends itself to the likelihood that lenalidomide crosses the blood-retinal barrier to alter local intraocular immunity which can lead to CMV reactivation [8]. While no consensus exists, there is some evidence to support that the drug can modulate the blood-retinal barrier which typically protects the eye from bloodstream pathogens [9].

Some differences between the diagnostic work-up and management of these two cases exist. Unlike the case presented by Lim and colleagues, our patient first received an anterior chamber paracentesis which was negative. Vitreous sample was obtained surgically via pars plana vitrectomy as opposed to a vitreous tap. Although both approaches were successful in identifying a causative organism, our approach allowed for concurrent removal of vitreous debris and placement of prophylactic endolaser to the necrotic retina. This may prevent future retinal detachment, a devastating sequela of CMV retinitis that occurs in upwards of 30% of patients [10]. Our patient was treated only with oral valganciclovir versus intravenous ganciclovir. Both patients did well with a good visual outcome and no recurrence. We continued our patient on a maintenance dose of oral valganciclovir 900 mg po daily for the duration of his lenalidomide therapy.

This case represents the second report of CMV retinitis associated with lenalidomide, a relatively new drug that received FDA approval in 2013. Lenalidomide is a derivative of thalidomide and works by modulating the substrate specificity of the CRL4^CRBN^E3 ubiquitin ligase which leads to subsequent proteasomal degradation of certain transcription factors that kills multiple myeloma cells. It is more appropriate to consider lenalidomide an immunomodulator rather than an immunosuppressant because its use actually stimulates certain immunogenic cell lines (IL-2 production in T lymphocytes, natural killer cells). Its use in diseases like myelodysplastic syndromes and mantle cell lymphoma is growing, and it is possible that cases such as this one will be seen more frequently in upcoming years. Although CMV retinitis often carries a poor visual prognosis, its course can be halted with early diagnosis and treatment. Because CMV retinitis classically affects AIDS patients, providers may fail to consider this diagnosis in the types of patients treated with lenalidomide. However, it seems that even immunocompetent HIV-negative patients can be affected by CMV retinitis in the context of lenalidomide treatment. Thus, it is critical that patients being treated with lenalidomide receive prompt evaluation if they develop ophthalmic symptoms.

## Additional Points

*Summary Statement.* A relatively immunocompetent patient with multiple myeloma being treated with lenalidomide presented with cytomegalovirus (CMV) retinitis. This report supports an association between lenalidomide and CMV retinitis and offers a different management approach from the one previously reported in a similar case.
